# Intimate partner violence during pregnancy and adverse birth outcomes in Ethiopia: A systematic review and meta-analysis

**DOI:** 10.1371/journal.pone.0275836

**Published:** 2022-12-22

**Authors:** Habtamu Gebrehana Belay, Getachew Arage Debebe, Alemu Degu Ayele, Bekalu Getnet Kassa, Gedefaye Nibret Mihretie, Lealem Meseret Bezabih, Mulugeta Dile Worke

**Affiliations:** 1 Department of Midwifery, College of Health Sciences, Debre Tabor University, Debre Tabor, Ethiopia; 2 Department of Nursing, College of Health Sciences, Debre Tabor University, Debre Tabor, Ethiopia; 3 School of Medicine, College of Health Sciences, Debre Tabor University, Debre Tabor, Ethiopia; Children’s Hospital of Eastern Ontario (CHEO), University of Ontario, CANADA

## Abstract

**Background:**

Intimate partner violence is a significant public health issue that affects maternal and neonatal health worldwide. Several studies have been conducted to investigate the prevalence of intimate partner violence during pregnancy as well as the factors that contribute to it. As a result, the purpose of this study was to determine the impact of intimate partner violence on birth outcomes.

**Methods:**

International databases including Scopus, PubMed, Google Scholar, Embase, and CINAHL were used to search primary studies. The quality and strength of the included studies were evaluated using the Newcastle-Ottawa Scale quality assessment tool. The studies heterogeneity and publication biases were assessed using I^2^ statistics and Egger’s regression test. The Meta-analysis was carried out using STATA version 16 software.

**Results:**

A total of nine hundred and fifty-eight articles were retrieved from various databases, and seventeen articles were included in the review. The pooled prevalence of intimate violence during pregnancy in Ethiopia was 32.23% (95% CI 28.02% -36.45%). During pregnancy, intimate partner violence was a significant predictor of low birth weight (AOR: 3.69, 95%CI 1.61–8.50) and preterm birth (AOR: 2.23, 95%CI 1.64–3.04).

**Conclusion:**

One in every three pregnant women experiences intimate partner violence. Women who experienced intimate partner violence during their pregnancy are more likely to experience adverse outcomes such as premature delivery and low birth weight infants.

## Introduction

Intimate partner violence is a significant public health issue that affects people worldwide. This serious public health and human rights issue includes physical, sexual, and emotional abuse [1]. More than one in every three women has experienced psychological abuse, nearly 20% has experienced sexual violence, and 99% of women who have experienced IPV have experienced financial or economic abuse [2]. According to the World Health Organization (WHO), one out of every three women has been sexually violence during their lifetime [3].

Intimate partner violence(IPV) occurs in all settings and among all socioeconomic, religious, and cultural groups [4]. IPV is one of the most common forms of gender-based violence, and it can have both short and long-term negative consequences for babies and mothers [5]. Physical, psychological, emotional, sexual, economic, and social abuse are manifestations of IPV, which have been shown to have a negative impact on maternal health and adverse birth outcomes [6, 7]. Women who have been exposed to IPV are less likely to obtain adequate prenatal and skilled delivery care than women who have not been abused [8].

IPV is more common among women of childbearing age, and pregnancy is particularly vulnerable for women in terms of physical and mental health [9, 10]. IPV exposure during the perinatal period may increase the risk of premature birth (PTB), low birth weight (LBW), prolonged neonatal intensive care unit stays, and fetal death [11]. Despite being more common than other obstetric problems such as preeclampsia or gestational diabetes, women are not screened for IPV during pregnancy [12, 13]. IPV in pregnancy is associated with adverse pregnancy outcomes, including increased risk of human immunodeficiency virus infection, perinatal depression, uterine rupture, hemorrhage, maternal death, PTB, LBW, stillbirth, and insufficient weight gain in pregnancy [13–15]. IPV during pregnancy is also linked to severe complications, including miscarriage, premature rupture of membranes, placental abruption, placental previa, PTB, neonatal death, and postpartum hemorrhage [16]. Negative maternal behaviours, insufficient nutrition or prenatal care, and increased stress levels contribute to poor birth outcomes [13].

Women’s coping with the stress, shame, and suffering from IPV during pregnancy has been linked to a number of unhealthy behaviors, including smoking, alcohol and substance abuse, an unhealthy eating pattern, and less likely to seek health care [17, 18]. During pregnancy, IPV has been linked to higher levels of depression, anxiety, stress, suicide attempts, a lack of attachment to the child, and lower breastfeeding rates [19, 20].

IPV prevalence in pregnancy has been reported to vary depending on the definition used [21], the measurement strategy [22], and the socio-cultural context of the population studied [12]. IPV prevalence in pregnancy varies across countries, with one-quarter of mothers worldwide exposed to IPV [23]. In Ethiopia, the prevalence of IPV during pregnancy ranges from 20.6% in the Tigray region [24] to 44.5% in the Oromia region [25]. According to a multilevel analysis of the 2016 Ethiopian demographic and health survey report, IPV prevalence was 28.74% [26]. In Ethiopia, there were inconsistencies in the prevalence of IPV and the adverse birth outcomes associated with IPV during pregnancy. This study aimed to estimate the pooled prevalence of IPV during pregnancy and its impact on birth outcomes in Ethiopia.

## Methods

PRISMA guidelines were followed in conducting the review and reporting the findings of this systematic review and meta-analysis [27].

### Inclusion and exclusion criteria

This review included primary studies on IPV during pregnancy conducted in Ethiopia between 2012 and March 12, 2022. Studies that did not show either at least one adverse pregnancy outcome or the prevalence of IPV during pregnancy were excluded.

### Measurement of the outcome variables

The outcome variables were the prevalence of IPV during pregnancy and adverse birth outcomes associated with IPV during pregnancy.

### Data sources and search strategy

Grey literature deposited on the websites of universities and research institutes and published studies in Ethiopia were searched. Articles for this systematic review were found using electronic web-based searches on PubMed, Google Scholar, Scopus, Embase, and CINAHL. Our search strategy included key terms and phrases such as “intimate partner violence”, “adverse birth outcome," "domestic violence during pregnancy," "effect of intimate partner violence during pregnancy," "prevalence of intimate partner violence," and “Ethiopia.” The following search strategy was used in the advanced PubMed database: (effect[All Fields] AND ("intimate partner violence"[MeSH Terms] OR ("intimate"[All Fields] AND "partner"[All Fields] AND "violence"[All Fields]) OR "intimate partner violence"[All Fields]) AND ("pregnancy"[MeSH Terms] OR "pregnancy"[All Fields])) AND ("pregnancy complications"[MeSH Terms] OR ("pregnancy"[All Fields] AND "complications"[All Fields]) OR "pregnancy complications"[All Fields] OR ("adverse"[All Fields] AND "birth"[All Fields] AND "outcomes"[All Fields]) OR "adverse birth outcomes"[All Fields]) AND ("Ethiopia"[MeSH Terms] OR "Ethiopia"[All Fields]). Then, using PubMed, we found 644 articles (S1 File).

### Data extraction and quality assessment

Three independent reviewers extracted the data using a structured data extraction form. The data extraction tool includes the primary author’s name, the year of publication, the study setting, sample size, study design, the prevalence of intimate violence during pregnancy, and adverse birth outcomes. Uncertainties during the extraction process were resolved by involving the fourth and fifth authors. A Newcastle-Ottawa Scale quality assessment tool was used to assess the quality and strength of the included studies [28]. Two reviewers (HG and AD) were independently assessed to check the quality of the included studies. Primary studies with a score of 8 were considered high quality, while those with a score of 6–7 were considered moderate quality and were included in the systematic review and meta-analysis.

### Data synthesis and analysis

For data entry and statistical analysis, the STATA-16 software was used [29]. We measured outcomes using estimates of adjusted odds ratios with confidence intervals (CI) in the meta-analysis. The pooled estimate of the prevalence of IPV during pregnancy and its adverse birth outcomes was estimated using a random-effect model.

### Publication bias and heterogeneity

The I^2^ test statistic was used to examine the heterogeneity of the included studies. Heterogeneity was declared at p ≤ 0.05 [30]. A weighted DerSimonian and Laird random-effects model was used for pooled analysis [31].The Egger’s regression test was used to determine the statistical significance of publication bias, and a p-value of less than 0.05 was used to declare the statistical significance of publication bias [32]. The Duval and Tweedie nonparametric trim and fill analysis accounted for publication bias in studies that showed it was present.

### Study selection

**Fig 1:**
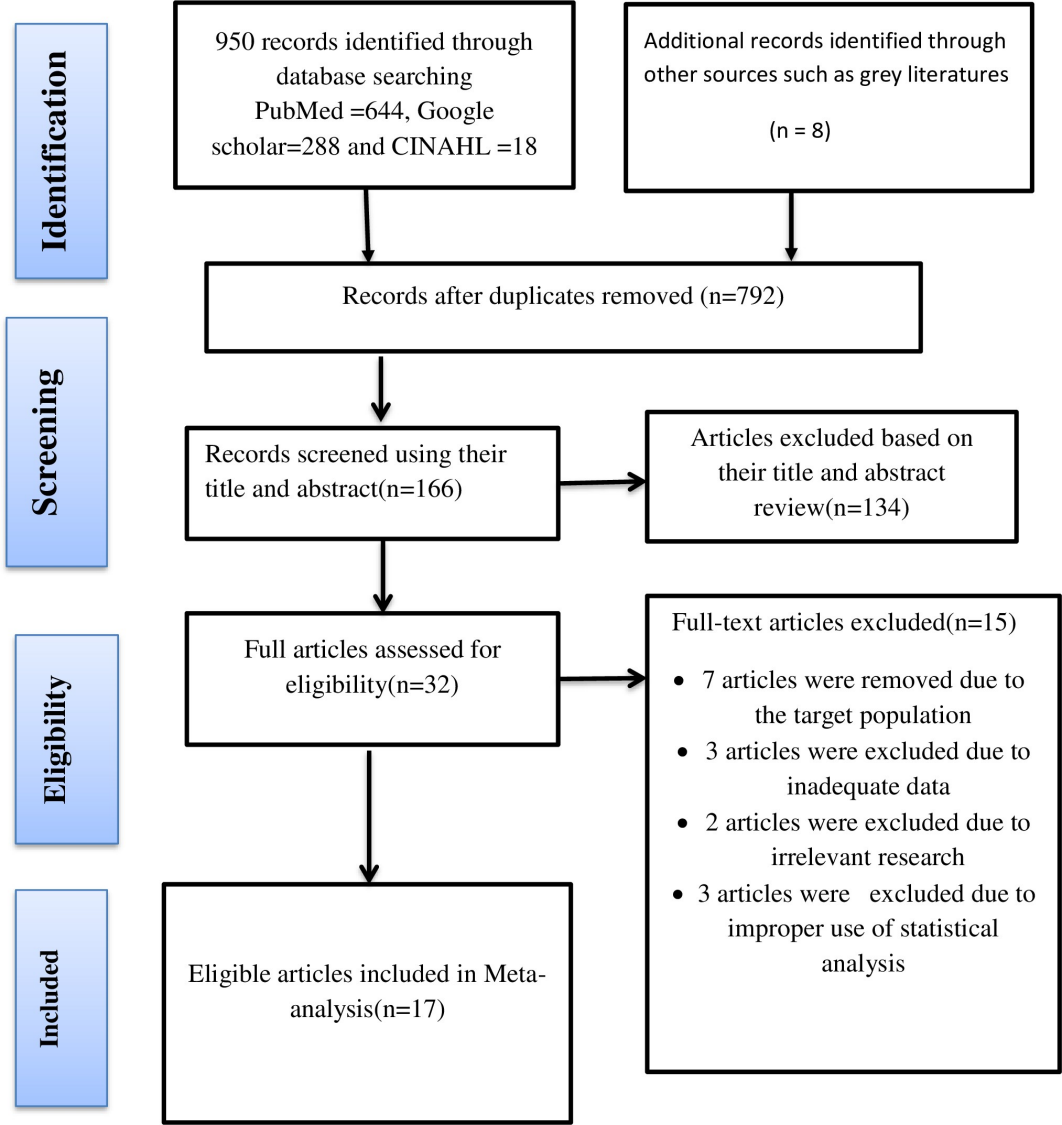
PRISMA flow chart for selection of studies publication.

A total of 958 primary studies were identified using data-driven searching. Seven hundred ninety-two articles were removed due to duplication, 134 articles were excluded based on a review of their title and abstract, and 15 full-text articles were excluded due to the target population, insufficient data, irrelevant research, and improper use of statistical analysis. Three independent reviewers screened the title and abstract, and potentially relevant articles were chosen for full-text review, and seventeen articles were chosen for the systematic review and meta-analysis. The disagreement between the reviewers was resolved using established article selection criteria (Fig 1)

## Results

### Characteristics of the included studies

This review included 17 studies with a total sample size of 10736. The sample size ranged from 195 in the Southern Nations, Nationalities, and Peoples’ Region (SNNPR) [33], to 3015 in the Harar town, Eastern Ethiopia [34]. There were fourteen cross-sectional studies and three case-control studies. From reviewed articles, four studies (23.5%) were from the Amhara region, four studies (23.5%) were from the Tigray region, three studies (17.6%) were from Southern Nations, Nationalities, and Peoples’ Region (SNNPR), two studies (11.7%) were from Harar town, one study (5.9%) were from Addis Ababa and three studies (17.6%) were from Oromia region (Table 1).

**Table 1:** Characteristics of 17 included studies in the meta-analysis of IPV and adverse birth outcomes, Ethiopia 2022.

Author	Year of publication	Region	Study design	Outcome	Sample size	Prevalence(%)
Berhanie et al.	2019	Tigray	Case-control	LBW, PTB	954	-
Laelago et al.	2017	SNNPR	Cross-sectional	LBW	195	23
Demelash et al.	2015	Oromia	Cross-sectional	LBW	408	25.8
Musa et al.	2021	Harar	Cross-sectional	LBW,PTB	603	39
Tadesse et al.	2020	Amhara	Case-control	PTB	414	-
Deyessa et al.	2016	Addis Ababa	Case-control	LWB	366	-
Ashenafi et al.	2020	Harar	Cross-sectional	-	3015	30.5
Fetene et al.	2022	SNNPR	Cross-sectional	-	594	39.2
Abebe Abate et al.	2016	Oromia	Cross-sectional	-	299	44.5
Belay et al.	2019	SNNPR	Cross-sectional	-	606	21
Azene et al.	2019	Amhara	Cross-sectional	-	418	41.1
Atsbaha et al.	2022	Tigray	Cross-sectional	-	323	36.1
Gebrezgi et al.	2017	Tigray	Cross-sectional	-	422	20.6
Gashaw et al.	2018	Oromia	Cross-sectional	-	720	35.6
Yimer et al.	2014	Amhara	Cross-sectional	-	434	32.2
Bifftu et al.	2017	Amhara	Cross-sectional	-	422	25.4
Adhena et al.	2020	Tigray	Cross-sectional	-	543	37.5

**Keys;** LBW: Low Birth Weight, PTB: Preterm Birth

### Prevalence of intimate partner violence during pregnancy

**Fig 2:**
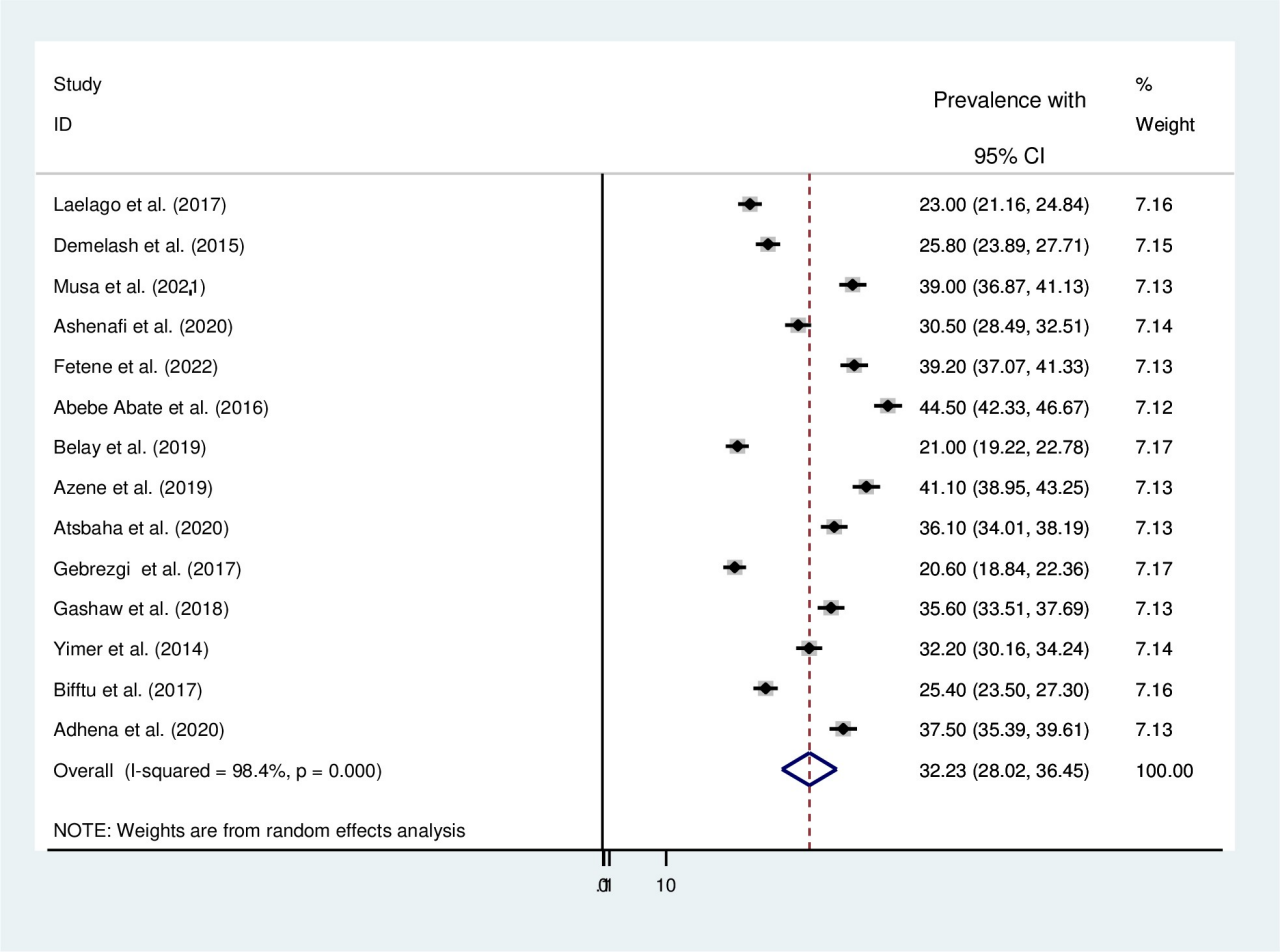
Forest plot for the pooled prevalence of intimate partner violence during pregnancy in Ethiopia.

In included studies, the prevalence of IPV during pregnancy was between 20.6% in the Tigray region [24] to 44.5% in the Oromia region [25]. The three studies from the Amhara region showed that the prevalence of IPV during pregnancy was 25.4%, 32.2%, and 41.1% [35–37]. The three studies from the Tigray region revealed that the prevalence of IPV during pregnancy was 20.6%, 36.1%, and 37.5% [24, 38, 39]. The three studies from the Oromia region revealed that the prevalence of IPV during pregnancy was 25.8%, 35.6%, and 44.5% [25, 40, 41]. The two studies from the Harar region revealed that the prevalence of IPV during pregnancy was 30.5% and 39% [34, 42], and the three studies from the SNNPR region revealed that the prevalence of IPV during pregnancy were 21%, 23%, and 39.2% [33, 43, 44]. The overall pooled prevalence of IPV during pregnancy was 32.23% (95% CI: 28.02% -36.45%)(Fig 2).

### Heterogeneity and publication bias

**Fig 3:**
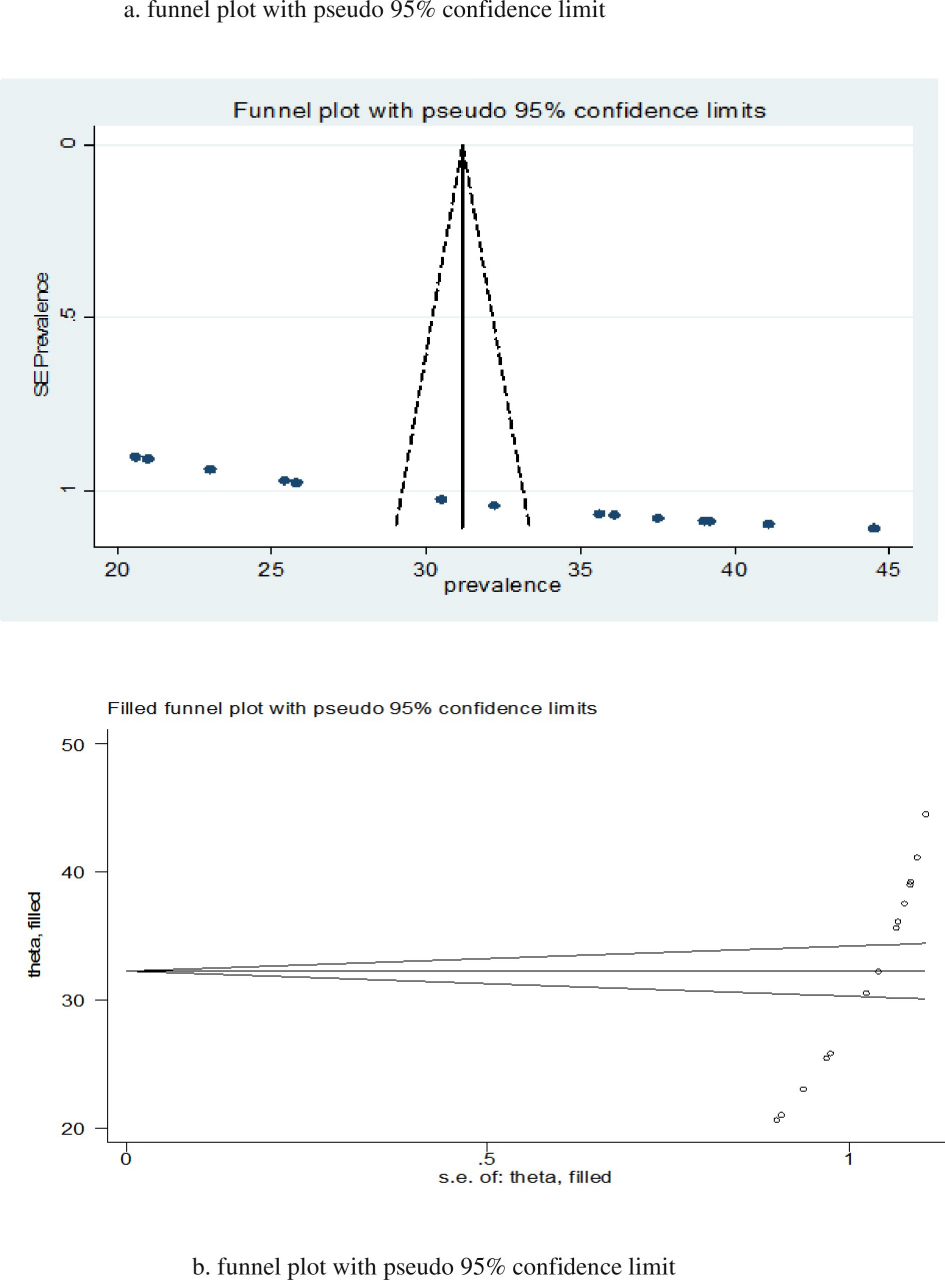
(a) funnel plot was used to examine the publication bias of 14 studies. b) No additional studies were added to the trim and fill analysis to correct publication bias.

The fourteen studies included in this meta-analysis to pool the prevalence of IPV during pregnancy revealed high heterogeneity, as evidenced by the I ^2^ test (I^2^ = 98.4%; p-value < 0.001). A funnel plot and the Egger’s regression test were used to assess publication bias. The p-value for Egger’s regression test was ≤ 0.001, indicating the presence of publication bias across studies. A funnel plot revealed asymmetrical distribution for this review (Fig 3A). The Duval and Tweedie nonparametric trim and fill analysis was used to correct publication bias among the 14 included studies reporting IPV prevalence during pregnancy. However, no studies were imputed for missing studies in trim and fill analysis (Fig 3B).

### Sensitivity analysis

A sensitivity analysis was performed using a random-effects model to identify outliers in the impact of a single study on the overall meta-analysis outcome. The sensitivity analysis revealed that no study fell outside the 95% confidence interval.

### Outcomes of intimate partner violence during pregnancy

**Fig 4:**
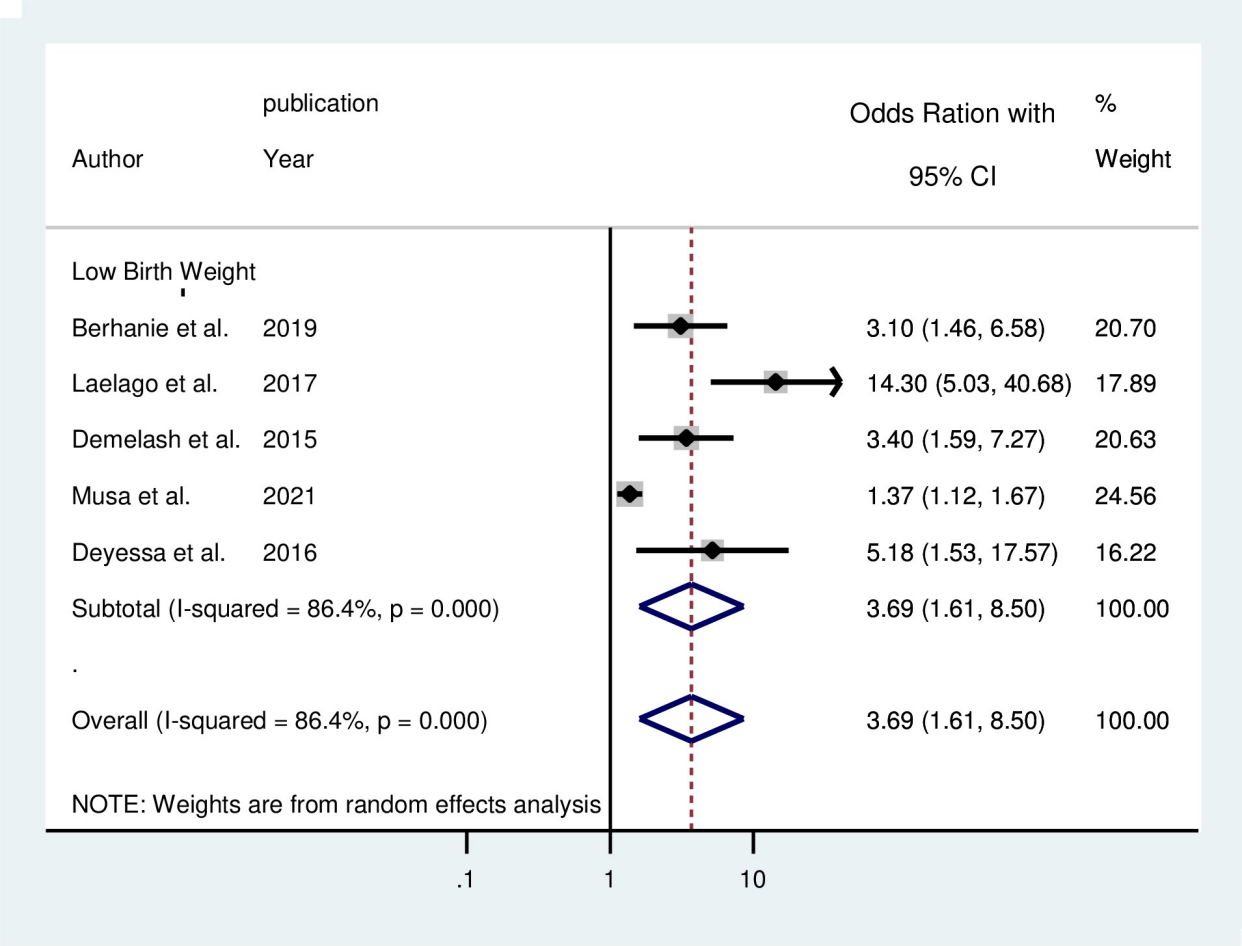
Forest plot for the pooled association between intimate partner violence during pregnancy and low birth weight.

**Fig 5:**
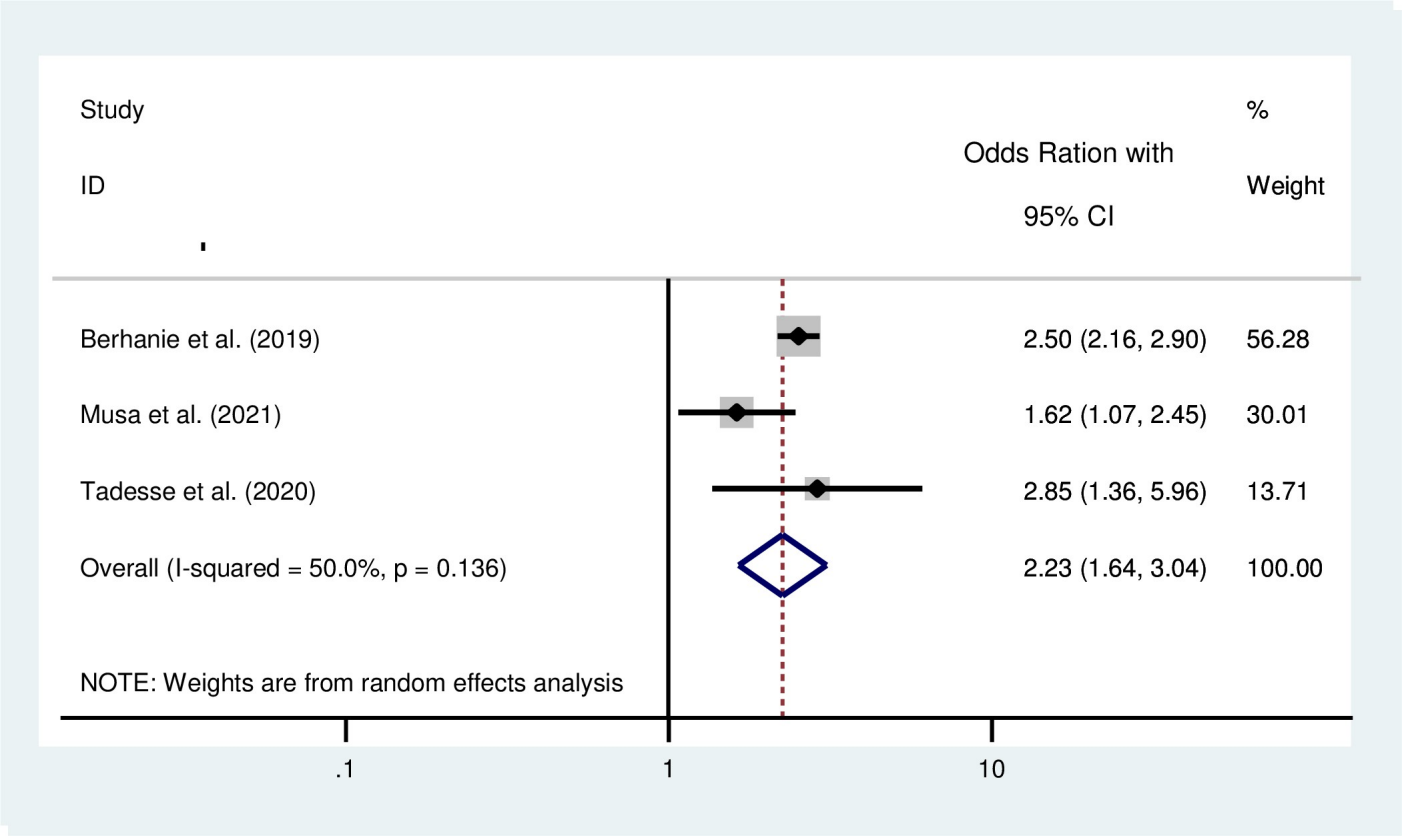
Forest plot for the pooled association between intimate partner violence during pregnancy and preterm birth in Ethiopia.

The pooled effect of five studies [33, 41, 42, 45, 46] revealed that IPV during pregnancy nearly quadruples the risk of low birth weight babies compared to women who were not exposed to IPV during pregnancy (AOR = 3.69; 95% CI: 1.61–8.50) (Fig 4). The pooled effect of three studies [42, 46, 47] revealed that IPV during pregnancy increased the risks of preterm birth by two times as compared to those counterparts (AOR = 2.23; 95% CI: 1.64–3.04) (Fig 5).

## Discussion

This systematic review and meta-analysis aimed to investigate the effect of IPV during pregnancy on birth outcomes. According to this systematic review and meta-analysis, the national pooled prevalence of IPV during pregnancy in Ethiopia is 32.23% (95% CI 28.02% -36.45%). Similar findings in Kenya revealed that the overall prevalence of IPV was 34.1% [48], and 35% in Vietnam [49]. and One-third (36%) of women in low- and middle-income countries(LMICs) had experienced emotional, physical, or sexual IPV during pregnancy [50].

This result is higher than the previous global systematic review and meta-analysis result of 25%, which implies that one-quarter of all mothers worldwide had been exposed to IPV during pregnancy [22]. This is also significantly higher than a systematic review and meta-analysis review in China, which found a pooled prevalence of IPV during pregnancy was 7.7% [51]. This could be due to differences in the geographical area, living conditions, and social, economic, and educational levels. Several studies demonstrated huge inequalities in IPV levels across LMICs. Poorer, younger, and less empowered women are particularly vulnerable to IPV exposure in most countries [14, 52, 53]. IPV is generally higher in LMICs than in high-income countries [14, 54].

This study revealed that IPV exposure during pregnancy was a major risk factor that nearly quadrupled the likelihood of having a baby with low birth weight. This conclusion is supported by a global systematic review and meta-analysis report on IPV during pregnancy and the risk of adverse infant outcomes [5, 55]. Other research has found that maternal exposure to IPV is significantly associated with an increased risk of low birth weight [56, 57]. This review finding is consistent with studies conducted in South Africa [58], Bangladesh [59], and Nigeria [60]. This could be because physical, sexual, and psychological violence against women during pregnancy causes despair, anxiety, and stress. This may impact pregnancy physiology and feeding habits, resulting in poor birth outcomes.

This systematic review and meta-analysis showed that exposure to IPV during pregnancy was associated with a 2-fold increase in the risks of preterm birth. This finding has been consistent with other systematic reviews and meta-analyses revealed that women who reported experiencing any IPV during pregnancy are more likely to deliver preterm than their non-abused counterparts across different settings [5, 13, 61]. This review finding is also consistent with studies conducted in Peru [62], Tanzania [63], Vietnam [64], and Zimbabwe [65]. This might be because maternal physical or sexual abuse has been linked to placental abruption, uterine contractions, premature membrane rupture, and genitourinary infections, all of which can lead to preterm birth. Violence may influence behavioural risk factors such as drug use, smoking, poor nutrition, inadequate prenatal care, or late entry to prenatal care, all of which contribute to preterm birth [66, 67]. Malnourished pregnant women had twofold higher odds of preterm birth than their healthy counterparts, and preterm birth can be caused by maternal stress via physiological and behavioural pathways [68, 69].

## Conclusion

In Ethiopia, IPV is a major public health issue, affecting one out of every three pregnant women. During pregnancy, intimate partner abuse was associated with adverse birth outcomes, particularly low birth weight and premature birth. Effective policies and initiatives promoting women’s empowerment, education, and development are needed to address the issue of IPV against women. Gender equality will be promoted by developing interventions to prevent violence against women and safe and responsible partnerships. This review will be used to provide up-to-date information for policymakers, health care providers, planners, researchers, and program managers. It will also serve as a platform for identifying current gaps that will necessitate the implementation of additional interventions. Strengthening the initiation of antenatal care is essential because it provides a window of opportunity for screening, treatment, and management of pregnant women who may be subjected to violence.

## Supporting information

S1 File A searching strategy for intimate partner violence during pregnancy and adverse birth outcomes. (DOCX)

S2 File Data. (XLSX)

S3 File Prisma checklist. (DOCX)
