# Supplementary material for: Intimate partner violence during pregnancy and adverse birth outcomes in Ethiopia: A systematic review and meta-analysis
Source: PLoS One. 2022 Dec 22;17(12):e0275836. doi: 10.1371/journal.pone.0275836 (PMC9778523; doi:10.1371/journal.pone.0275836)
Supplement: S1 File — (DOCX) [file pone.0275836.s001.docx]

**Additional file 1:** Searching strategy for intimate partner violence during pregnancy and adverse birth outcomes: A systematic review and meta-analysis

| Databases | Searching terms | Number of studies |
| --- | --- | --- |
| PubMed | (effect[All Fields] AND ("intimate partner violence"[MeSH Terms] OR ("intimate"[All Fields] AND "partner"[All Fields] AND "violence"[All Fields]) OR "intimate partner violence"[All Fields]) AND ("pregnancy"[MeSH Terms] OR "pregnancy"[All Fields])) AND ("pregnancy complications"[MeSH Terms] OR ("pregnancy"[All Fields] AND "complications"[All Fields]) OR "pregnancy complications"[All Fields] OR ("adverse"[All Fields] AND "birth"[All Fields] AND "outcomes"[All Fields]) OR "adverse birth outcomes"[All Fields]) AND ("ethiopia"[MeSH Terms] OR "ethiopia"[All Fields]) | 644 |
| Google scholar | The effect of intimate partner violence during pregnancy and adverse birth outcomes | 288 |
| HINARI | The effect of intimate partner violence during pregnancy and adverse birth outcomes | 18 |
| Others databases |  | 8 |
| Total retrieved |  | 958 |
| Included |  | 17 |
